# Cytokine rs361525, rs1800750, rs1800629, rs1800896, rs1800872, rs1800795, rs1800470, and rs2430561 SNPs in relation with prognostic factors in acute myeloid leukemia

**DOI:** 10.1002/cam4.2424

**Published:** 2019-08-01

**Authors:** Claudia Bănescu, Florin Tripon, Adrian P. Trifa, Andrei G Crauciuc, Valeriu G. Moldovan, Alina Bogliş, Istvan Benedek, Delia Dima, Marcela Cândea, Carmen Duicu, Mihaela Iancu

**Affiliations:** ^1^ Genetics Laboratory, Center for Advanced Medical and Pharmaceutical Research University of Medicine, Pharmacy, Science and Technology of Târgu Mureș Târgu Mureș Romania; ^2^ Department of Medical Genetics University of Medicine, Pharmacy, Science and Technology of Târgu Mureș Târgu Mureș Romania; ^3^ Department of Medical Genetics ”Iuliu Hatieganu” University of Medicine and Pharmacy Cluj‐Napoca Romania; ^4^ Department of Internal Medicine University of Medicine, Pharmacy, Science and Technology of Târgu Mureș Târgu Mureș Romania; ^5^ Department of Hematology The Oncology Institute Prof. Dr.I. Chiricuta Cluj Napoca Romania; ^6^ Department of Clinical Science University of Medicine, Pharmacy, Science and Technology of Târgu Mureș Târgu Mureș Romania; ^7^ Department of Medical Informatics and Biostatistics ”Iuliu Hatieganu” University of Medicine and Pharmacy Cluj‐Napoca Romania

**Keywords:** acute myeloid leukemia, cytokine, gene polymorphisms, mortality, prognostic factors

## Abstract

**Background:**

Cytokines were correlated with survival and disease progression in acute myeloid leukemia (AML). We aimed to evaluate the multivariate effect of *TNF‐α* rs361525, rs1800750, rs1800629, *IL‐10* rs1800896, rs1800872, *IL‐6* rs1800795, *TGF‐β1* rs1800470, *IFN‐γ* rs2430561 single nucleotide polymorphisms (SNPs) on AML risk, the multivariate effect of SNPs on overall survival (OS) in AML and the association between the investigated SNPs and prognostic factors in AML.

**Methods:**

All SNPs were genotyped in 226 adult AML cases and 406 healthy individuals. AML patients were investigated for *FLT3* (ITD, D835), *DNMT3A* (R882), and *NPM1* type A mutations.

**Results:**

Univariate analysis revealed that age above 65 years had a negative influence on survival (*P* < .001). The presence of the rs1800750 variant genotype (*P* = .005) or *FLT3‐ITD* mutation (*P* = .009) in a cytogenetic high‐risk group (*P* = .003) negatively influenced OS. A negative association was observed between Eastern Cooperative Oncologic Group Scale status > 2, lactate dehydrogenase (LDH) level, platelet (PLT) count <40 000 cells/mm^3^, and OS. Multivariate Cox regression analysis showed that the presence of the rs1800750 variant genotype was a risk factor for death (*P* = .007), and that blast percentage, LDH level (≥600 IU/L), and cytogenetic high‐risk were independent significant predictors for death in AML (*P* = .04, corrected HR = 1.20; *P* = .022, corrected HR = 1.24; *P* = .021, corrected HR = 1.34, respectively).

**Conclusions:**

Age above 65 years, PLT count, *TNF‐α* rs1800750 variant genotype, blast percentage, LDH level, and cytogenetic high‐risk may be used as independent risk factors to assess AML mortality.

## Graphical abstract

We aimed to evaluate the multivariate effect of TNF‐α rs361525, rs1800750, rs1800629, IL‐10 rs1800896, rs1800872, IL‐6 rs1800795, TGF‐β1 rs1800470, IFN‐γ rs2430561 single nucleotide polymorphisms (SNPs) on AML risk, the multivariate effect of SNPs on overall survival (OS) in AML and the association between the investigated SNPs and prognostic factors in AML. A negative association was observed between Eastern Cooperative Oncologic Group Scale status >2, lactate dehydrogenase (LDH) level, platelet (PLT) count <40 000 cells/mm3, and OS. Ages above 65 years, PLT count, TNF‐α rs1800750 variant genotype, blast percentage, LDH level, and cytogenetic high‐risk may be used as independent risk factors to assess AML mortality.

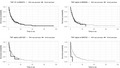

## 1 INTRODUCTION

Acute myeloid leukemia (AML) is a complex and dynamic human malignancy characterized by multiple somatically‐acquired driver mutations, poor prognosis, and short survival, less than 20% of adult AML patients surviving 5 years after diagnosis.^1^, ^2^

Cytokines (interleukins [ILs], growth factors, interferons, etc) play an important role in regulating the inflammatory response, and chronic inflammation and are involved in cancer development.^3^, ^4^ Chronic inflammation is associated with the release of various mediators (pro‐inflammatory and oncogenic ones), such as reactive nitrogen oxygen species, inflammatory cytokines (IL‐1β, IL‐2, IL‐6, and tumor necrosis factor alpha [TNF‐α]), growth factors, and chemokines.^4^

Based on their inflammatory activity, cytokines are divided into pro‐inflammatory (IL‐6, IL‐17, IL‐18, TNF‐α, interferon gamma [IFN‐γ], etc) 5 and anti‐inflammatory (Il‐4, IL‐10, IL‐13, etc) ones, whereas several cytokines have a dual role (IL‐10, IL‐22, TGF‐β1).^6^ Interleukin‐10, an anti‐inflammatory cytokine, has dual functions, being both immune‐suppressive (tumor‐inhibiting) and immune‐stimulating (tumor‐promoting), and may, therefore, influence tumor susceptibility and development. Transforming growth factor beta (TGF‐β) regulates normal hematopoiesis, which is frequently disrupted in hematologic malignancies. The most common alteration in hematologic malignancies is the development of resistance to TGF‐β homeostatic functions such as proliferation, differentiation, and apoptosis.^7^

Aberrant cytokine production was hypothesized to play a substantial role in the pathogenesis of cancer and several hematological malignancies, including myeloproliferative neoplasms (MPN) and AML.^8^

Disturbances in proinflammatory (IL‐1, IL‐2, IL‐6, IL‐8, IL‐12, TNF‐α, and IFN‐γ) and anti‐inflammatory (IL‐4, IL‐10, etc) cytokines, as well as different growth factors, confirm the existence of an inflammatory reaction associated with MPNs that may trigger disorder initiation and lead to the development of myelofibrosis.^9^

A recent study revealed that inflammatory cytokines play a critical role in the expansion of leukemic cells and AML progression, demonstrating the promoting effect and functional relevance of the aberrant production of IL‐1 cytokines in the pathobiology of AML.^10^

Cytokine plasma levels have been correlated with survival, event‐free survival, and disease progression in AML cases.^8^

Single nucleotide polymorphisms (SNPs) have been found in cytokine genes, suggesting that certain alleles could lead to variations in cytokine production capacity.^11^, ^12^ Therefore, it could be hypothesized that cytokine gene variants may influence gene expression and plasma levels and could, therefore, be associated with the pathogenesis of hematological malignancies.

The association between the *IFN‐γ* +874T>A (rs2430561) polymorphism and the risk of chronic myeloid leukemia (CML)^13^ or chronic lymphocytic leukemia (CLL) was previously evaluated.^14^ A recent meta‐analysis suggested that the *IFN‐γ* +874T>A polymorphism contributes to CML and CLL susceptibility.^15^ Currently, studies investigating patients with AML and cytokine polymorphisms involvement are infrequent.

In a previous study, no association was found between *TGF‐β1* rs1800470 polymorphism and leukemia.^16^ In contrast, Nursal et al showed that variants of *TNF‐α* 238G>A rs361525, *IL‐10* (−1082G>A rs1800896, −819C>T rs1800871, −592C>A rs1800872), and *TGF‐β1* (codon 25) genes may have a significant association with AML etiopathogenesis.^17^ However, no association was observed between *TNF‐α* −308G>A, *IL‐10* (−592T>G, −819T>C, −1082T>C), *IFN‐γ* +874T>A and *TGF‐β1* (codons 10 and 25) polymorphisms and the risk of CML.^13^

Carcinogenesis is affected by the action of several genes, in which an isolated SNP plays only a small role. Therefore, it is necessary to evaluate more than one SNP in the same study to take into account the polygenic model of inherited cancer susceptibility.^18^

To the best of the authors' knowledge, there are no published studies describing the possible association between cytokine polymorphisms and biological parameters, overall survival (OS), and AML prognostic impact.

The objectives of this study were to evaluate: (a) the multivariate effect of eight SNPs [*TNF‐α* 238G>A (rs361525); 376G>A (rs1800750); 308G>A (rs1800629)], *IL‐10* −1082T>C (rs1800896); −592T>G (rs1800872), *IL‐6* 174C>G (rs1800795), *TGF‐β1* 869C>T (rs1800470), *IFN‐γ* +874T>A (rs2430561)] on AML risk; (b) the multivariate effect of the eight SNPs on OS in AML patients; (c) the association between studied polymorphisms and clinical prognostic factors in AML patients.

## 2 MATERIAL AND METHODS

### 2.1 Patients and controls

The AML cases were comprised of 226 adults from the Central and North‐Eastern regions of Romania diagnosed with AML at the Hematology Clinics from Targu Mures and the Hematology Department of The Oncology Institute “Prof. Dr Ion Chiricuta” in Cluj‐Napoca, Romania.

For comparison, a control group of 406 healthy Romanian non‐related individuals from the same geographic region was included in the study. These healthy individuals had no history of malignancy and had been initially referred to the Emergency County Hospital from Targu Mures for the investigation of anemia or leukocytosis.

All participants signed written informed consent. The study was approved (No. 67 from 14 April 2017) by the Board of the Ethical Committee of the University of Medicine and Pharmacy of Targu Mures and was carried out in conformity with the principles of the Helsinki Declaration.

According to the World Health Organization (WHO) 2016 classification,^19^ the AML cases were categorized as follows: 67 AML cases with recurrent genetic abnormalities (29.6), 32 AML cases with myelodysplasia‐related changes (14.2%), no AML cases with myeloid sarcoma (0%), no AML cases with Down syndrome‐related myeloid proliferations (0%), four AML cases with therapy‐related myeloid neoplasms (1.8%), and 123 AML cases not otherwise specified (54.4%).

### 2.2 Genotyping

Quick‐gDNA MiniPrep kits (Zymo Research), Wizard Genomic DNA Purification kits (Promega), and PureLink Genomic DNA Mini Kits (ThermoFisher Scientific) were used for DNA isolation from fresh whole blood samples collected in Ethylenediaminetetraacetic acid (EDTA) tubes.

TaqMan technology was used for SNP genotyping. Pre‐designed TaqMan SNP Genotyping assays for *TNF‐α* rs361525 and rs1800750, and *IL‐10* rs1800896 and rs1800872, were used on a 7500 Fast Dx Real*‐*time PCR System.

*TNF‐α* rs1800629, *IL‐6* rs1800795, *TGF‐β1* rs1800470, and *IFN‐γ* rs2430561 were analyzed using the ARMS‐PCR method.^20^

*FLT3* (ITD, D835), *DNMT3A* (R882), and *NPM1* type A (c.863_864insTCTG) mutations were analyzed as previously reported.^21^, ^22^, ^23^, ^24^ Fragment analysis of *FLT3* ITD and *NPM1* type A mutation was performed in all cases and allow us to establish the ratio of mutated to normal alleles. High resolution melting (in‐house method) was used to re‐genotype 10% of the AML cases for *FLT3* and *DNMT3A* mutations. CastPCR was used for *NPM1* c.863_864insTCTG mutation analysis, using ThermoFisher assay numbers Hs00000953_mu and Hs00001029_rf.

The Ensembl genome browser (release version 93) was used to designate wild‐type and variant alleles for the investigated SNPs.^25^

### 2.3 Statistical analysis

Continuous variables with a Gaussian distribution were presented using statistics as the mean ± standard deviation (SD), whereas deviations from the normal probability law were described by the median and interquartile range (Q_1_; Q_3_). The distributions of the nominal variables were presented as absolute frequencies with percentages. Binomial logistic regression was used to test and quantify the impact of genetic and clinical factors on AML risk. Odds ratios with a 95% confidence interval (95% CI) were estimated.

The distribution of OS time was presented using medians with their 95% CI for the studied groups. In order to quantify the multivariate effects of all studied SNPs on OS, we used Cox regression, the full model tested consisting in eight SNPs (rs361525, rs1800750, rs1800629, rs1800896, rs1800872, rs1800795, rs1800470, and rs2430561 SNPs), four somatic mutations (*FLT3*‐ITD, *FLT3* D835, *NPM1*, *DNMT3A* R882), and 12 demographic and clinical determinants (age group, gender, platelet count [PLT], blasts [%], hematocrit, hemoglobin and lactate dehydrogenase [LDH] level, Eastern Cooperative Oncologic Group Scale (ECOG) performance status, cytogenetic risk with two dummy variables, white blood cell [WBC] count, AML type).

Due to the large set of possible predictors for OS in AML patients (eight SNPs, four somatic mutations, and 12 demographic and clinical determinants) and an EPV (number events per predictor) equal to 7 (EPV = 7), we used the Minimizing approximated Information Criterion (MIC) as a new sparse estimation method to select a set of predictors for OS in AML patients.^26^, ^27^ Those predictors of the full model that did not significantly contribute to the OS were excluded and a reduced model obtained by the MIC estimation method was presented.

The sample size of patients used for the Cox regression analysis was 204 cases with 170 events. The MIC estimation method retained seven out of 24 candidate predictors for OS in AML patients. The effect size of selected predictors on OS in the reduced model was described by corrected HR determined from the shrinkage estimators of regression coefficients and adjusted HR determined from classical estimators.

Cox proportional hazards regression analysis with parameters estimated via the MIC method was performed using the “coxphMIC” package in the R statistical computing environment.

For the haplotype analysis of *IL‐10* (rs1800896 and rs1800872) and *TNF‐α* (rs361525, rs1800629, and rs1800750) SNPs, we used the Haplotype Analysis^28^ free software.

## 3 RESULTS

### 3.1 Description of AML and control groups

Demographic data, as well as clinical and biological features of our AML patients, are presented in Table 1. Most AML cases were included in the intermediate cytogenetic risk group, as ECOG performance status grade 3 was observed in about 40% of investigated patients and most of them received a high dose of chemotherapy.

**Table 1:** Characteristics of AML patients

Variables	Number (%)
Age	
≤20 y	5 (2.2)
21‐40 y	47 (20.8)
41‐60 y	79 (35.0)
60‐90 y	95 (42.0)
Gender	
Female	113 (50.0)
Men	113 (50.0)
WBC (cells/mm^3^)	11 270.0 [31 00.0; 38 700.0]^a^
<50 000	183 (81.0)
≥50 000	43 (19.0)
PLT (cells/mm^3^)	36 500 [11 000.0; 77 000.0]^a^
<40 000	119 (52.7)
≥40 000	107 (47.3)
Hgb (g/dL)	8.73 [7.8; 10.2]^a^
<10	166 (73.5)
≥10	60 (26.5)
LDH level (IU/L)	688.5 [461.0; 1132.0]^a^
<600	96 (42.5)
≥600	130 (57.5)
Blasts (in bone marrow, %)	60.0 [40.0; 78.0]^a^
<70	138 (61.1)
≥70	88 (38.9)
Cytogenetic risk	
Low risk (favorable prognostic)	24 (10.6)
Intermediate	123 (54.4)
High risk (adverse)	57 (25.3)
Not available (NA)	22 (9.7)
ECOG performance status	
0	3 (1.3)
1	36 (15.9)
2	37 (16.4)
3	91 (40.3)
4	59 (26.1)
ECOG performance status	
≤1	39
≥2	187
AML subtype	
de novo AML	182 (81.5)
Secondary AML	40 (17.7)
Therapy related AML	4 (1.8)
*FLT3* status (ITD+D835)	
Absent	182 (81.4)
Present	42 (18.6)
*FLT3*‐ITD status	
Absent	191 (84.5)
Present	35 (15.5)
*FLT3* D835 status	
Absent	214 (94.7)
Present	12 (5.3)
*DNMT3A* R882 status	
Wild‐type	200 (88.5)
Mutant	26 (11.5)
*NPM1* c.863_864insTCTG status	
Wild‐type	189 (83.6)
Mutant	37 (16.4)
Treatment	
HD	119 (52.7)
LD	91 (40.3)
HD + HSCT	16 (7.0)

Abbreviations: AML, acute myeloid leukemia; ECOG, Eastern Cooperative Oncologic Group Scale; HD, high dose of chemotherapy; HSCT, hematopoietic stem cell transplantation; LD, low dose of chemotherapy; LDH, lactate dehydrogenase; PLT, platelet; WBC, white blood cell.

a Median and interquartile range (percentile 25%; percentile 75%).

The mean age ± SD of the AML groups was 54.44 ± 16.78 years (range 19‐87 years old), whereas the mean age ± SD for the controls was 56.01 ± 15.567 years (range 20‐85 years old). The control group included 213 females (52.5%) and 193 males (47.5%), whereas the AML group was made up of 113 (50%) females and 113 (50%) males. Age and gender distributions were similar in both the AML and controls (*P* ≥ .05). Seventy‐four AML patients (74/226, 32.7%) were ≥65 years of age.

### 3.2 Investigated SNPs and AML risk

The genotype distribution for all investigated *TGF‐β1, IFN‐γ, TNF‐α, IL‐6*, and *IL‐10* cytokine SNPs in AML patients and controls are presented in Table 2. None of the *IFN‐γ* rs2430561, *IL‐10* (rs1800872, rs1800896) and *TNF‐α* (rs361525, rs1800629, rs1800750), genotypes demonstrated deviation from the Hardy‐Weinberg equilibrium (HWE) in either the AML cases or controls. *TGF‐β1* rs1800470 and *IL‐6* rs1800795 genotypes were not consistent with HWE (*P* < .05) in AML patients but were in HWE on controls. The *TGF‐β1* rs1800470 heterozygous genotype was associated with AML development risk and *IFN‐γ* rs2430561 heterozygous genotype was a protective factor for AML (OR = .63, 95% CI: 0.42‐0.94, *P* = .024), while the other studied SNPs were not associated with AML risk. Regarding allele distribution, no differences were observed between the investigated groups except for the variant A allele of *TNF‐α* rs1800750 (*P* = .002).

**Table 2:** Genotype of the investigated cytokine gene polymorphisms in AML patients and healthy controls

SNP	Control group (n_1_ = 406)	AML group (n_2_ = 226)	COR (95% CI)	*P* a	AOR (95% CI)	*P* b
*TGF‐β1* rs1800470						
Additive model	**n (%)** c	**n (%)** d				
GG	50 (12.3)	16 (7.1)	Ref.		Ref.	
GA	165 (40.6)	135 (59.7)	2.56 (1.39‐4.69)	**.002**	2.53 (1.38‐4.65)	**.003**
AA	191 (47.0)	75 (33.2)	1.23 (0.66‐2.29)	.520	1.20 (0.65‐2.26)	.550
Dominant model						
AA	50 (12.3)	16 (7.1)	Ref.		Ref.	
GA + AA	356 (87.7)	210 (92.9)	1.84 (1.02‐3.32)	**.042**	1.82 (1.01‐3.29)	**.046**
*IFN‐γ* rs2430561						
Additive model						
TT	67 (16.5)	54 (23.9)	Ref.		Ref.	
TA	215 (53.0)	105 (46.5)	0.61 (0.40‐0.93)	**.022**	0.61 (0.40‐0.94)	**.024**
AA	124 (30.5)	67 (29.6)	0.67 (0.42‐1.07)	.092	0.67 (0.42‐1.07)	.092
Dominant model						
TT	67 (16.5)	54 (23.9)	Ref.		Ref.	
TA + AA	339 (83.5)	172 (76.1)	0.63 (0.42‐0.94)	**.024**	0.63 (0.42‐0.95)	**.026**
*TNF‐α* rs361525						
Additive model						
GG	389 (95.8)	211 (93.4)	Ref.		Ref.	
GA	17 (4.2)	15 (6.6)	1.63 (0.77‐3.32)	.182	1.09 (0.79‐1.52)	.207
*TNF‐α* rs1800629						
Additive model						
GG	271 (66.7)	148 (65.5)	Ref.		Ref.	Ref.
GA	127 (31.3)	73 (32.3)	1.05 (0.74‐1.50)	.775	1.05 (0.74‐1.49)	.778
AA	8 (2.0)	5 (2.2)	1.14 (0.37‐3.56)	.816	1.14 (0.38‐3.74)	.756
Dominant model						
GG	271 (66.7)	148 (65.5)	Ref.		Ref.	
GA + AA	135 (33.3)	78 (34.5)	1.06 (0.75‐1.49)	.748	1.06 (0.75‐1.50)	.739
*TNF‐α* rs1800750						
GG	402 (100.0)	220 (97.3)	Ref.	**.002**	Ref.	Ref.
GA	0 (0.0)	6 (2.7)	ND		ND	ND
*IL‐6* rs1800795						
CC	68 (16.7)	32 (14.2)	Ref.		Ref.	
CG	188 (46.3)	127 (56.2)	1.44 (0.89‐2.31)	.137	1.43 (0.89‐2.30)	.143
GG	150 (36.9)	67 (29.6)	0.95 (0.57‐1.58)	.841	0.93 (0.56‐1.55)	.782
CG + GG	338 (83.3)	194 (85.8)	1.22 (0.77‐1.92)	.393	1.21 (0.79‐1.52)	.417
*IL‐10* rs1800872						
GG	222 (54.7)	117 (51.8)	Ref.		Ref.	Ref.
GT	158 (38.9)	99 (43.8)	1.19 (0.85‐1.67)	.314	1.21 (0.86‐1.70)	.271
TT	26 (6.4)	10 (4.4)	0.73 (0.34‐1.57)	.418	0.73 (0.34‐1.56)	.410
GT + TT	184 (45.3)	109 (48.2)	1.12 (0.81‐1.56)	.482	1.14 (0.82‐1.58)	.435
*IL‐10* rs1800896						
TT	144 (35.5)	74 (32.7)	Ref.		Ref.	Ref.
TC	188 (46.3)	109 (48.2)	1.13 (0.78‐1.63)	.519	1.12 (0.77‐1.61)	.560
CC	74 (18.2)	43 (19.0)	1.13 (0.71‐1.81)	.607	1.11 (0.69‐1.77)	.677
TC + CC	262 (64.5)	152 (67.3)	1.13 (0.80‐1.59)	.490	1.11 (0.79‐1.57)	.545

Abbreviations: AOR, adjusted odd ratio for age and gender; CI, confidence interval; COR, crude odd ratio.

a *P*‐value obtained from Chi‐square test, Fisher's Exact test or univariate binomial logistic regression.

b *P*‐value obtained from multivariable logistic model.

c Percentages were calculated for genotypes relative to the number of controls.

d Percentages were calculated for genotypes relative to the number of cases.

Bold values denoted statistically significant results (*P* < 0.05).

We analyzed whether there were any differences between the investigated groups in the presence of at least two variant genotypes and observed that the presence of four variant genotypes from the investigated SNPs were associated with AML risk with a tendency towards statistical significance (*P* = .059, OR = 1.45, 95% CI: 0.99‐2.13).

### 3.3 Bivariate associations between studied SNPs and clinical factors

The clinical features of AML patients according to the presence of wild‐type and variant genotypes for each cytokine polymorphism are presented in Table 3. No significant associations were observed between investigated SNPs and age when we divided our cases into three groups (≤40 years, 41‐60 years, ≥61 years). A significant difference in frequencies was found only in the case of *IL‐10* rs1800872 variant genotype in patients <40 years vs patients ≥40 years of age (*P* = .03), with a higher frequency of the variant genotype in elderly patients. There was an association between age ≥65 years and *IL‐10* rs1800896 (*P* = .019).

**Table 3:** Patients' characteristics according to the *TGF‐β1* rs1800470, *IFN‐γ* rs2430561, *TNF‐α* rs361525, rs1800629, rs1800750, *IL‐10* rs1800896, rs1800872, *IL‐6* rs1800795 genotypes

	*TGF‐β1* rs1800470			*IFN‐γ* rs2430561			*TNF‐α* rs361525				*TNF‐α* rs1800629			*TNF‐α* rs1800750				*IL‐6* rs1800795			*IL‐10* rs1800872			*IL‐10* rs1800896		
	GG	GA + AA	*P*‐value	TT	TA + AA	*P*‐value	GG	GA + AA	*P*‐value		GG	GA + AA	*P*‐value	GG	GA + AA		*P*‐value	CC	CG + GG	*P*‐value	GG	GT + TT	*P*‐value	TT	TC + CC	*P*‐value
Age groups																										
19‐40 y	3	49	.208	12	40	.369	47	5	.199	35		17	.821	50	2	.438		9	43	.623	21	31	.101	18	34	.108
41‐60 y	3	46		23	56		72	7		53		26		76	3			9	70		47	32		19	60	
61‐90 y	10	85		19	76		92	3		60		35		94	1			14	81		49	46		37	58	
Gender																										
Female	12	101	**.038**	30	83	.349	106	7	.789	76		37	.576	109	4	.683		17	96	.849	57	56	.690	33	80	.257
Male	4	109		24	89		105	8		72		41		111	2			15	98		60	53		41	72	
WBC (cells/mm^3^)																										
<50 000	13	170	.977	41	142	.279	172	11	.453	113		72	**.015**	180	3	.085		25	158	.658	93	90	.555	61	122	.697
≥50 000	3	40		13	10		39	4		35		8		40	3			7	36		24	19		13	30	
PLT (cells/mm^3^)																										
<40 000	9	110	.765	28	91	.892	108	11	.097	78		41	.984	114	5	.216		16	103	.745	62	57	.916	39	80	.992
≥40 000	7	100		26	81		103	4		70		37		106	1			16	91		55	52		35	72	
Hgb (g/dL)																										
<10	13	153	.646	39	127	.815	153	13	.23	114		52	.094	161	5	1.00		25	141	.518	89	77	.356	53	113	.664
≥10	3	57		15	45		58	2		34		26		59	1			7	53		28	32		21	39	
LDH level (IU/L)																										
<600	6	90	.676	20	76	.354	92	4	.20	53		43	**.005**	95	1	.244		10	86	.165	47	49	.467	30	66	.681
≥600	10	120		34	96		119	11		95		35		125	5			22	108		70	60		44	86	
Blasts (in bone marrow, %)																										
<70	12	126	.236	29	109	.204	129	9	.930	89		49	.694	134	4	1.00		19	119	.833	73	65	.671	39	99	**.072**
≥70	4	84		25	63		82	6		59		29		86	2			13	75		44	44		35	53	
AML subtype																										
de novo	13	169	.75	44	138	.75	168	14	.483	123		59	.071	176	6	.636		28	154	**.03**	93	89	.897	63	199	.517
sAML	3	37		10	30		39	1		21		19		40	0			2	38		22	18		10	30	
tAML	0	4		0	4		4	0		4		0		4	0			2	2		2	2		1	3	
Cytogenetic risk																										
Low‐risk	1	23	.153	7	17	.649	22	2	.121	10		14	.083	24	0	.184		5	19	.441	12	12	.409	7	17	**.007**
Intermediate	13	110		32	91		117	6		84		39		121	2			17	106		59	64		43	80	
High‐risk	1	56		11	46		54	3		39		18		55	2			9	48		35	22		11	46	
NA	1	21		4	18		18	4		15		7		20	2			1	21		11	11		13	9	
ECOG status																										
≤1	4	35	.153	10	29	.778	36	3	.728	20		19	**.044**	37	2	.277		3	36	.203	19	20	.675	13	26	.931
≥2	12	175		44	143		175	12		128		59		183	4			29	158		98	89		61	126	

Abbreviations: sAML, secondary AML; tAML, therapy related AML; NA, not available.

Bold values denoted statistically significant results (*P* < 0.05).

A higher frequency in females was observed for *TGF‐β1* rs1800470 variant genotype (Table 3). We observed an association between WBC and *TNF‐α* rs1800629, the variant genotype being higher in patients with WBC <50 000/mm^3^ than in patients with WBC >50 000/mm^3^ (*P* = .015).

Furthermore, a higher frequency of the *TNF‐α* rs1800629 variant genotype was found in patients with LDH <600 IU/L (*P* = .005; OR = 0.45; 95% CI: 0.26‐0.80).

No associations were observed between blast percentage and investigated SNPs, but there was a tendency towards statistical significance between blasts <70% and *IL‐10* rs1800896 (*P* = .072).

Regarding AML subtypes, we found an association between the *IL‐6* rs1800795 variant genotype (*P* = .03) and a higher frequency of variant genotypes between the AML de novo cases, with a trend towards statistical significance (*P* = .071) in the case of *TNF‐α* rs1800629 SNP.

Regarding the relationship between cytogenetic risk groups and cytokine gene polymorphisms, there was a significant association in the case of *IL‐10* rs1800896 variant genotype (*P* = .007) and a trend towards statistical significance (*P* = .083) in case of *TNF‐α* rs1800629 for high cytogenetic risk.

We observed an association between each ECOG performance status grade and the variant genotypes of *IFN‐γ* rs2430561 (*P* = .043), *TNF‐α* rs1800750 (*P* = .005) while *TNF‐α* rs1800629 variant genotype was associated with ECOG grade ≥2 (*P* = .044).

Regarding the association between somatic mutations and investigated cytokines SNPs, the presence of *TNF‐α* rs1800629 variant genotype was associated with *FLT3‐ITD* (*P* = .049), *FLT3* (ITD+D835) (*P* = .048), and *DNMT3A* somatic mutations (*P* = .008), whereas the *FLT3*‐D835 or *NPM1* type A mutations showed no association (*P* > .05).

We also tested the association between somatic mutations and combinations of 3, 4, 5, or 6 variant genotypes in the studied SNPs, but only the presence of five variant genotypes with the *FLT3*‐ITD mutation (*P* = .081) and six variant genotypes with the *NPM1* mutation (*P* = .081) showed a trend towards statistical significance.

There was not found significant associations between studied SNPs and type of treatment (*P* > .05) on all AML cases (n = 226).

### 3.4 The individual impact of studied SNPs, demographic and clinical factors on OS time

There was an association between AML patients' age and survival (*P* < .001). Patients under 65 had a better survival than patients over 65 [median survival time, 95% CI: 9.0 (7.4‐10.6) vs 3.0 (2.1‐3.9)], whereas gender and AML subtype showed no difference (*P* = .523). On the other hand, there was a significant difference in survival curves distributions (*P* < .001) between low, intermediate, and high cytogenetic risk groups [median survival time, 95% CI: 12.0 (6.3‐17.7); 7.0 (5.1‐8.9), and 3.0 (1.4‐4.6)].

We observed a better outcome in AML patients with an ECOG performance status ≤1 compared to those with a status ≥2 (*P* = .001, median survival time, 95% CI: 12.0 (6.9‐17.1) vs 6.0 (4.4‐7.6)). Survival time showed a significant difference (*P* < .001) in AML patients treated with high‐dose (HD) chemotherapy (median survival time, 95% CI: 9.0 [6.9‐11.0]), low‐dose (LD) chemotherapy (median survival time, 95% CI: 4.0 [3.3‐4.7]), and HD chemotherapy + hematopoietic stem cell transplantation (HSTC) (median survival time, 95% CI: 12.0 [9.1‐14.9]).

Patients with WBC >50 000/mm^3^ at diagnosis had a median survival time of 7 months vs 5 months for patients with WBC <50 000/mm^3^ at diagnosis (*P* = 0.053).

Kaplan‐Meier analysis showed that AML patients with PLT >40 000/mm^3^ at diagnosis had an increased rate of survival compared to those with lower PLT levels (Log‐Rank test, *P* = .05). When we analyzed the univariate impact of LDH level on OS, we observed that LDH levels above 600 IU/L were significantly associated with a shorter OS (*P* = .003, median survival time, 95% CI: 5.0 [3.7‐6.3] vs 10.0 [6.8‐13.2)].

**Figure 1:**
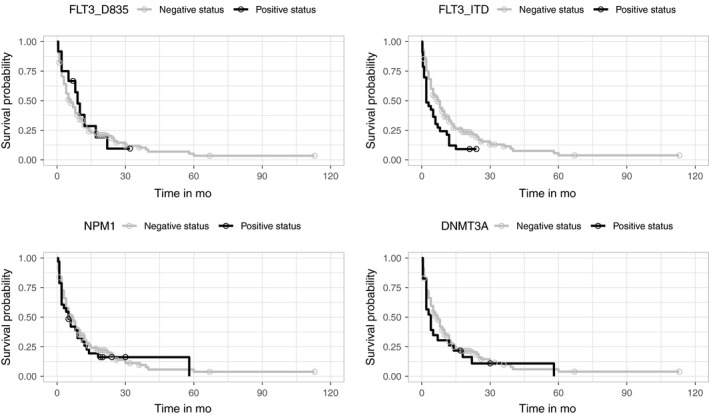
The Kaplan‐Meier curves for *FLT3*‐D835, *FLT3*‐ITD, *NPM1* and *DNMT3A* of all patients included in survival analysis. Circles marks indicated censored cases

Patients with the *FLT3‐ITD* mutation had a significantly shorter OS (*P* = .004) than those with *FLT3‐ITD* negative status (median survival time, 95% CI: 2.0 [0.3‐3.7] vs 7.0 [5.2‐8.8]). The Kaplan‐Meier curves depicted in Figure 1 showed the percentage of survival in the AML cases with *FLT3‐ITD* positive and negative status. Moreover, in AML patients with *FLT3* mutation, the presence of the *D835* mutation did not affect OS (*P* = .689). When we analyzed *FLT3* (ITD+D835) positive status, we observed a trend towards statistical significance for this association (*P* = .078) with survival. No association was found between *NPM1* or *DNMT3A* status, hemoglobin, hematocrit levels, and bone marrow blast percentage at diagnosis and OS (*P* > .05).

**Figure 2:**
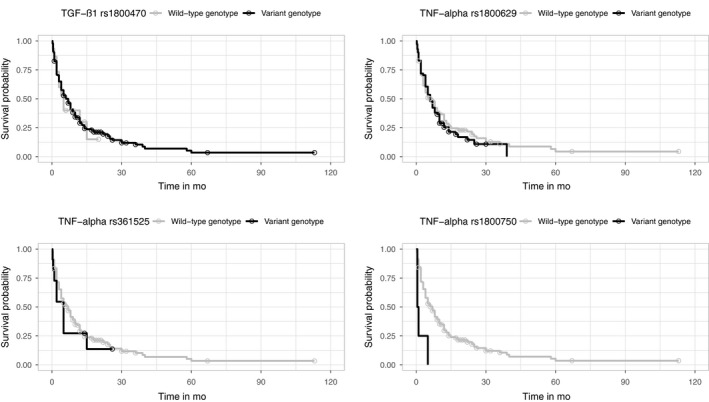
The Kaplan‐Meier curves for *TGF‐β1* rs1800470, *TNF‐α* rs361525, *TNF‐α* rs1800629 and *TNF‐α* rs1800750 of all patients included in survival analysis. Circles marks indicated censored cases

**Figure 3:**
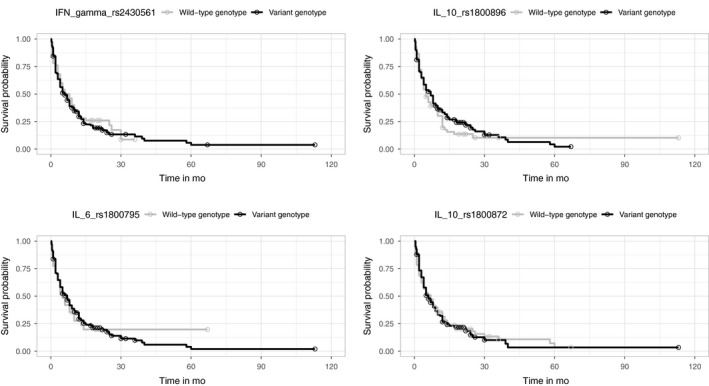
The Kaplan‐Meier curves for *IFN‐γ* rs2430561, *IL‐6* rs1800795, *IL‐10* rs1800896 and *IL‐10* rs1800872 of all patients included in survival analysis. Circles marks indicated censored cases

The *TNF‐α* (rs361525, rs1800750, and rs1800629), *IL‐10* (rs1800896 and rs1800872), *IL‐6* (rs1800795), *TGF‐β1* (rs1800470), and *IFN‐γ* (rs2430561) variant genotypes were not associated with patients' OS (*P* > .05), and neither were the combined variant genotypes (presence of >3 variant genotypes). Kaplan‐Meier curves were plotted to show the crude survival rates for the eight SNPs. (Figures 2 and 3).

### 3.5 The size effect of studied SNPs and clinical factors on mortality

The effects of the studied SNPs on OS in AML patients were assessed by Cox regression analysis (univariable and multivariable). Patients lacking cytogenetic risk group data were not included in this analysis, therefore only 204 AML cases were investigated, with death occurring in 170 of these patients.

The results of the Cox proportional hazards regression regarding the effects of the studied cytokine SNPs on mortality in AML patients are presented in Table 4.

**Table 4:** The results of the Cox proportional hazards regression: unadjusted and adjusted effects of the studied SNP on overall survival in AML patients

Factors	Levels/genotypes	Events (%)	Univariable analysis		Multivariable analysis‐reduced model		
			HR^a^ (95% CI)	*P*‐value	HR^b^ (95% CI)	Corrected^c^ HR (95% CI)	*P*‐value
Gender	F	79 (80.6)	Reference	.293	NA	NA	NA
	M	91 (85.8)	1.18 (0.87‐1.59)				
Age	<65 y	109 (79.0)	Reference	**<.001**	Reference	Reference	**<.001**
	≥65 y	61 (92.4)	**2.15 (1.55**‐**2.99)**		**2.76 (1.96**‐**3.89)**	**1.61 (1.37**‐**1.89)**	
*TGF‐β1* rs1800470	Wild‐type genotype	11 (73.3)	Reference	.857	NA	NA	NA
	variant	159 (84.1)	0.95 (0.51‐1.75)				
*IFN‐γ* rs2430561	Wild‐type genotype	41 (82.0)	Reference	.705	NA	NA	NA
	variant	129 (83.8)	1.07 (0.75‐1.53)				
*TNF‐α* rs361525	Wild‐type genotype	161 (83.4)	Reference	.546	NA	NA	NA
	variant	9 (81.8)	1.23 (0.63‐2.41)				
*TNF‐α* rs1800629	Wild‐type genotype	111 (83.5)	Reference	.500	Reference	Reference	.070
	variant	59 (83.1)	1.12 (0.81‐1.54)		1.39 (0.99‐1.93)	1.17 (1.00‐1.38)	
*TNF‐α* rs1800750	Wild‐type genotype	166 (83.0)	Reference	**.005**	Reference	Reference	**.007**
	variant	4 (100.0)	**4.22 (1.54**‐**11.52)**		**5.84 (2.07**‐**16.45)**	**1.27 (1.10**‐**1.47)**	
*IL‐6* rs1800795	Wild‐type genotype	24 (77.4)	Reference	.941	NA	NA	NA
	variant	146 (84.4)	0.98 (0.64‐1.52)				
*IL‐10* rs1800872	Wild‐type genotype	88 (83.0)	Reference	.885	NA	NA	NA
	variant	82 (83.7)	1.02 (0.76‐1.38)				
*IL‐10* rs1800896	Wild‐type genotype	52 (85.2)	Reference	.385	NA	NA	NA
	variant	118(82.5)	0.87 (0.62‐1.20)				
*FLT3*‐ITD status	Absent	140 (81.9)	Reference	**.009**	NA	NA	NA
	Present	30 (90.9)	**1.70 (1.14**‐**2.54)**				
*FLT3* D835 status	Absent	160 (83.3)	Reference	.739	NA	NA	NA
	Present	10 (83.3)	0.90 (0.47‐1.70)				
*NPM1* status	Absent	142 (83.0)	Reference	.618	NA	NA	NA
	Present	28 (84.8)	1.11 (0.74‐1.66)				
*DNMT3A* R882 status	Absent	149 (82.3)	Reference	.368	NA	NA	NA
	Present	21 (91.3)	1.23 (0.78‐1.95)				
Hgb level	<10	127 (82.5)	Reference	.217	NA	NA	NA
	≥10	43 (86.0)	0.80 (0.57‐1.14)				
Htc	>35	11 (100.0)	Reference	.851	NA	NA	NA
	≤35	159 (82.4)	0.94 (0.51‐1.74)				
PLT	<40 000	93 (84.5)	**1.40 (1.03**‐**1.89**)	**.039**	1.39 (0.99‐1.85)	1.17 (1.00‐1.38)	.075
	≥40 000	77 (81.9)	Reference		Reference	Reference	
Blasts (%)	<70	98 (79.0)	Reference	.325	Reference	Reference	**.042**
	>70	72 (90.0)	1.17 (0.86‐1.58)		**1.46 (1.06**‐**2.03)**	**1.20 (1.03**‐**1.41)**	
LDH level (IU/L)	<600	68 (78.2)	Reference	**.008**	Reference	Reference	**.022**
	≥600	102 (87.2)	**1.52 (1.11**‐**2.06)**		**1.54 (1.10**‐**2.16)**	**1.24 (1.05**‐**1.47)**	
ECOG status	≤1	18 (56.3)	Reference	**.010**	NA	NA	NA
	≥2	152 (88.4)	**1.90 (1.16**‐**3.10)**				
Cytogenetic risk (1)	Low‐risk	17 (70.8)	Reference	.422	NA	NA	NA
	Intermediate	98 (79.7)	1.24 (0.74‐2.07)				
Cytogenetic risk (2)	Low‐risk	17 (70.8)	Reference	**.003**	Reference	Reference	**.021**
	High‐risk	55 (96.5)	**2.29 (1.32**‐**3.97)**		**1.54 (1.10**‐**2.16)**	**1.34 (1.15**‐**1.56)**	
WBC count	<50 000	137 (82.5)	Reference	.077	NA	NA	NA
	≥50 000	33 (86.8)	1.41 (0.96‐2.07)				
AML type	0	137 (82.0)	Reference	.266	NA	NA	NA
	1 + 2	33 (89.2)	1.24 (0.85‐1.82)				

Events = number of deaths; % was calculated in relation to the number of cases corresponding to each category of the study.

Cytogenetic risk variable was transformed in two dummy variables in cox regression analysis

Abbreviations: 95% CI, 95% confidence interval; HR, hazard ratio; MIC, Minimizing approximated Information Criterion; NA, not selected to be included in the reduced model.

a Crude HR.

b Adjusted HR from unpenalized Cox PH regression model.

c Adjusted HR from penalized Cox PH regression model estimated by MIC method.

Bold values denoted statistically significant results (*P* < 0.05).

The univariate effect of investigated parameters on OS indicated that an age above 65 years had a significant negative influence on survival (*P* < .001, HR = 2.15, 95% CI: 1.55‐2.99). Presence of the *TNF‐α* rs1800750 variant genotype (*P* = .005; HR = 4.22, 95% CI: 1.54‐11.52), or the *FLT3*‐ITD mutation (*P* = .009; HR = 1.70; 95% CI: 1.14‐2.54), and inclusion in the cytogenetic high risk group (*P* = .003; HR = 2.29; 95% CI: 1.32‐3.97) negatively influenced OS. In the case of AML patients with a cytogenetic risk established (n = 204) the treatment type (HD, LD, HD + HSCT) was significantly associated with OS (Log‐Rank test, *χ* ^2^(2) = 25.7, *P* < .001), the risk of death was higher in those treated with HD than in patients with LD (crude HR = 2.18, 95% CI: 1.57‐3.02). In addition, the effects of cytokines on OS were not modified after controlling for the treatment effect, of all polymorphisms only *TNF‐α* rs1800750 SNP having a significant effect on OS after controlling treatment effect (stratified Cox regression, *P* = .0003, stratification variable: treatment type, adjusted HR = 5.84, 95% CI: 2.09‐16.31).

A negative association was observed between ECOG status with a grade ≥2, LDH level ≥600 IU/L, a PLT count lower than 40 000/mm^3^ and OS (Table 4).

Multivariate Cox regression analysis based on the MIC estimation method retained only seven predictors from the 24 candidate predictors for OS in AML patients (namely age, *TNF‐α* rs1800629 and rs1800750, PLT count, bone marrow blast percentage, LDH level, and cytogenetic high risk). Based on our Cox proportional hazards regression findings, we considered that age above 65 years was an independent predictor for mortality in AML cases (*P* < .001, HR = 1.61, 95% CI: 1.37‐1.89). Following multivariate analysis, we observed a trend towards statistical significance for the association between *TNF‐α* rs1800629 variant genotype, PLT count, and mortality (*P* = .07, corrected HR = 1.17, 95% CI: 1.00‐1.38; *P* = .075, corrected HR = 1.17, 95% CI: 1.00‐1.38).

The *TNF‐α* rs1800750 variant genotype was a risk factor for death (*P* = .007, corrected HR = 1.2, 95% CI: 1.10‐1.47). Furthermore, we observed that blast percentage, LDH level, and cytogenetic high‐risk were independent significant predictors for death in AML (*P* = .04, corrected HR = 1.20, 95% CI: 1.03‐1.41; *P* = .022, corrected HR = 1.24, 95% CI: 1.05‐1.47; *P* = .021, corrected HR = 1.34, 95% CI: 1.15‐1.56, respectively).

## 4 DISCUSSION

Our study provides data regarding the impact of cytokine gene polymorphisms in AML patients and AML mortality predictors in a Romanian case‐control study.

We found that the *TGF‐β1* rs1800470 and *IFN‐γ* rs2430561 variant genotypes were associated with AML susceptibility for the additive and dominant models. Furthermore, no evidence of association was observed between the investigated cytokine SNPs and AML in the allelic model, except for *TNF‐α* rs1800750 A allele (*P* = .002).

Regarding *TGF‐β1* rs1800470, our findings were similar to those of a study performed in Brazil which demonstrated that the *TGF‐β1* rs1800470 TT homozygous genotype was associated with an increased risk of developing hematological malignancies (OR = 4.07; 95% CI: 1.94‐8.52; *P* = .0002), with a 4‐fold increase in the risk of developing hematological cancers.^18^ Pehlivan et al found no significant differences between Turkish patients with Philadelphia positive CML and their controls.^13^ Contrary to our observations, no difference was observed in a Turkish population for the *TGF‐β1* rs1800470 variant genotype.^17^

Regarding *IFN‐γ* +874T>A (rs2430561), no association was detected between the variant genotype or variant allele and AML risk in Turkey.^17^ In the meta‐analysis performed by Wu et al, no significant association was found between *IFN‐γ* +874T>A polymorphism and leukemia risk for all comparison models. In the case of subgroup analysis by leukemia type, a significantly increased risk for CML was observed in the dominant model (TT + TA vs AA, OR = 1.783, 95% CI: 1.236‐2.573, *P* = .002) and a decreased risk for CLL was found in the allelic, co‐dominant, and dominant models when separately using the fixed‐effect model (T vs A, OR = 0.660, 95% CI: 0.483‐0.902, *P* = .009; TT vs AA, OR = 0.472, 95% CI: 0.247‐0.902, *P* = .023 and TT + TA vs AA, OR = 0.457, 95% CI: 0.285‐0.734, *P* = .001).^15^ The difference in these findings may be due to the ethnicity of the studied populations, and the number of AML cases evaluated.

The *TNF‐α* rs1800629, *IL‐10* rs1800872, and *IL‐10* rs1800896 SNPs were not risk factors for AML development in our population, with similar findings being reported by Pehlivan et al.^13^ Similarly, a meta‐analysis of 19 publications comprising 1509 patients with leukemia and 4075 controls found no association between the *TNF‐α* rs1800629 polymorphism and leukemia risk.^29^ In contrast, a recent study showed statistically significant differences regarding the genotype and allele distribution of *TNF‐α* rs1800629 in CLL patients compared to controls (*P* = .00003 and *P* = .00007).^30^

In addition, our findings indicated that *TNF‐α* rs361525 did not confer susceptibility to AML in the additive and dominant models. No studies are currently available regarding the role of rs361525 in AML susceptibility, however, a closely‐related hematological malignancy case‐control study (123 Brazilian patients with MPN and 123 healthy subjects) showed that the rs361525 and rs1800629 variant genotypes were significantly higher in MPN cases (*P* = .04 and *P* = .02), providing a risk factor for developing MPN of 2.21 and 1.82, respectively.^31^ The rs1800750 heterozygous genotype was associated with a higher risk for Hodgkin's lymphoma in Mexican patients (OR = 4.41 95% CI: 1.21‐16.6, *P* = .02).^32^ Similarly, in our study, the heterozygous genotype was found only in AML cases but not in controls, and we considered that rs1800750 heterozygous genotype may be associated with an AML risk (*P* = .002). In the allelic model, we noticed that *TNF‐α* rs1800750 A allele was associated with AML susceptibility (*P* = .002).

Furthermore, we observed no differences regarding the frequency of *IL‐6* rs1800795 genotypes or alleles among AML patients and controls, our data being similar to the results reported by Nursal et al.^17^ In contrast, the presence of the rs1800795 variant genotype could be associated with CML susceptibility in Turkish patients.^13^, ^33^

Research results regarding the role of *IL‐10* SNPs in the predisposition to leukemia are contradictory. The *IL‐10* −592C>A promoter SNP was investigated in 115 AML patients and 137 controls from China, where a significant difference regarding the −592AA genotype percentage (*P* = .014) and −592A allele frequency (*P* = .004) was observed. The −592AA genotype prevalence risk was 2.492 times higher than in CC genotype carriers (OR = 2.492; 95% CI: 1.013‐5.825).^34^ Moreover, a recent study reported that the presence of the *IL‐10* rs1800872 variant allele was associated with a slightly increased risk of AML (adjusted OR = 1.30 95% CI: 1.01‐1.72).^34^ Nursal et al showed that variants of the *IL‐10* (rs1800896 and rs1800872) gene may have a significant association with AML etiopathogenesis.^17^

Considering that no association could be detected between the *IL‐10* rs1800896 allele or genotype frequency and CML risk, a recent study indicated that *IL‐10* could be a useful survival biomarker in CML.^13^

In the present study, no relationship was found between *IL‐10* rs1800872 and rs1800896 and AML predisposition. These findings are in line with certain studies reported previously 13; however, they are also in contradiction with several others.^17^, ^34^, ^35^

Regarding *IL‐10* rs1800896, our findings were in line with those of Fei et al who found no significant differences between AML patients and controls when comparing rs1800896 allele and genotype frequencies.^35^ Hiroki et al observed no association between the *IL‐10* rs1800896 variant genotype and IL‐10 plasma levels and concluded that rs1800896 was not associated with acute lymphoblastic leukemia (ALL) susceptibility nor with relapse risk.^11^

We also investigated the potential association between the *IL‐10* and *TNF‐α* haplotypes and AML risk. The most frequent haplotypes of the *IL‐10* rs1800896 and rs1800872 SNPs were CTGG (23.5% patients vs 25.1% controls) and CTGT (24.3% patients vs 20.7% controls). Logistic regression did not confirm any significant association between CTGT haplotype and AML risk (*P* = .337, OR = 1.22, 95% CI: 0.81‐1.83), with similar results being found for the CTGG haplotype (*P* = .876, OR = 0.97, 95% CI: 0.65‐1.83).

We observed that the most frequent *TNF-α* haplotypes (rs361525, rs1800629, and rs1800750) were h5 AGGGGG haplotype (30.1% patients vs 30.3% controls) and GGGGGG (60.6% patients vs 63.5% controls), but neither was correlated with AML risk (GGGGGG haplotype *P* = .145, OR = 0.63, 95% CI: 0.34‐1.17; AGGGGG haplotype *P* = .208, OR = 0.66, 95% CI: 0.34‐1.26). Moreover, we analyzed the impact of the *IL‐10* and *TNF‐α* haplotypes on OS in our AML cohort. We did not find any differences in OS regarding the CTGG and CTGT haplotypes or any other *IL‐10* haplotype (log‐Rank test, *P* = .809), nor between AGGGGG and GGGGGG haplotypes and the other *TNF‐α* haplotypes (log‐rank test, *P* = .482).

The current study showed a significantly lower survival rate in elderly AML patients compared to patients under 65 years of age. A shorter OS was observed in AML cases with cytogenetic high risk, and in those with decreased WBC counts. High LDH (>600 IU/L) levels and *FLT3*‐ITD mutation negatively influenced overall AML survival. The investigated SNPs had no effect on OS in AML, either when they were tested individually or in the case of combined variant genotypes (presence of >3 variant genotypes from all eight). The fact that no associations were found between AML patients' OS and the presence of *TNF‐α* (rs361525, rs1800750, and rs1800629), *IL‐10* (rs1800896 and rs1800872), *IL‐6* (rs1800795), *TGF‐β1* (rs1800470), or *IFN‐γ* (rs2430561) variant genotypes suggests that these SNPs may not represent independent survival biomarkers in AML.

Moreover, in the univariate Cox regression analysis, we found that age above 65 years, *TNF‐α* rs1800750 variant genotype, *FLT3*‐ITD mutation, cytogenetic high risk, ECOG performance status ≥2, LDH level ≥600 IU/L, and PLT count lower than 40 000 cells/mm^3^ had an effect on OS in AML.

In multivariate Cox PH regression analysis, only age above 65 years, *TNF‐α* rs1800750 SNP, blast percentage, LDH level, and cytogenetic high‐risk were found to be independent significant predictors for OS in AML. We observed a trend towards statistical significance regarding the association between the *TNF‐α* rs1800629 variant genotype and OS. In contradiction, Kim et al reported a lower OS (estimated 20.1 months) in case of the GA variant genotype compared to GG homozygous genotype (estimated 54.6 months) for the *IL‐10* rs1800896 SNP in AML patients.^36^ Similar to our findings, Kim et al, revealed that the *IL‐10* rs1800871 and rs1800872 variant genotypes did not have an effect on OS.^36^ Similar results regarding this lack of effect on OS by *IL‐10* rs1800871 have been previously reported.^37^

Pehlivan et al found no association between *TNF‐α* rs1800629, *IL‐10* rs1800872, rs1800871, and rs1800896, *IFN‐γ* rs2430561, and *TGF‐β1* rs1800470 (codons 10 and 25) polymorphisms and OS in their Turkish CML patients,^13^ being therefore in line with our observations.

The relationship between any two combined variant genotypes of the *TGF‐β1, IFN‐γ*, *TNF‐α*, *IL‐6*, and *IL‐10* SNPs and AML risk was not evaluated because we only had a few cases with only one variant genotype and no cases with wild‐type homozygous genotype for all of the analyzed cytokine SNPs.

Our study contains a number of limitations, including the lack of data regarding cytokine expression and the lack of information regarding *RUNX1* and *CEBPA* mutations, the lack of data about the plasma level of TNF‐α, IL‐10, IL‐6, TGF‐β1 and IFN‐γ in the patient samples. However, the robustness of this study is represented by the fact that it investigated a representative AML cohort in Romania, since, to our knowledge, there is currently no published data on the role of these polymorphisms in AML on Eastern European populations. This is the first study to investigate the association between cytokine SNPs and AML risk, and, as a result, our report presents novel observations not previously described in a complex heterogeneous disease characterized by genomic changes and the accumulation of blasts, most commonly in the bone marrow, resulting in bone marrow failure and leading to death.^38^, ^39^

Furthermore, our study investigated, for the first time, the relationship between somatic mutations in AML and cytokine SNPs in a single multivariate model, in which the estimation method allowed the selection of variables, model fitting, and stable regression coefficients.

**In conclusion,** based on our findings, we consider that *TGF‐β1* rs1800470 and *IFN‐γ* rs2430561 variant genotypes were associated with AML susceptibility. Our study revealed that age above 65 years, PLT count (<40 000 cells/mm^3^), *TNF‐α* rs1800750 variant genotype, blast percentage (>70%), LDH level (≥600 IU/L), and cytogenetic high risk may be used as independent risk factors for assessing AML mortality.

## CONFLICT OF INTERESTS

The authors report no conflict of interests.
